# Implementing the Lung Donor (LUNDON) acceptability score in U.S. donor management and transplant decision-making: A multi-aim, mixed-methods protocol

**DOI:** 10.1371/journal.pone.0342383

**Published:** 2026-04-21

**Authors:** Nikki E. Rossetti, Samantha Morrison, Su-Hsin Chang, Yan Yan, Graham Jaensch-Frie, Charles R. Liu, Nahom Y. Seyoum, Meghna Katta, Arvind Kumar, Zhizhou Yang, Christian Oncken, Christy Hamilton, Nicholas Schroy, Kyle Stumbaugh, Gary Marklin, Brendan Heiden, Brendon Cummiskey, Sarah Peskoe, Matthew Hartwig, Varun Puri, Ana A. Baumann

**Affiliations:** 1 Division of Cardiothoracic Surgery, Department of Surgery, Washington University School of Medicine, St. Louis, Missouri, United States of America; 2 Department of Biostatistics & Bioinformatics, Duke University School of Medicine Durham, North Carolina, United States of America; 3 Division of Public Health Sciences, Department of Surgery, Washington University School of Medicine St. Louis, Missouri, United States of America; 4 Department of Surgery, Massachusetts General Hospital Boston, Massachusetts, United States of America; 5 United Network for Organ Sharing, Richmond, Virginia, United States of America; 6 Mid-America Transplant, St. Louis, Missouri, United States of America; 7 Division of Cardiovascular and Thoracic Surgery, Department of Surgery, Duke University School of Medicine Durham, North Carolina, United States of America; PLOS: Public Library of Science, UNITED KINGDOM OF GREAT BRITAIN AND NORTHERN IRELAND

## Abstract

**Background:**

Despite advances in transplantation science, a persistent shortage of donor lungs continues to limit access to life-saving lung transplants. The Lung Donor (LUNDON) Acceptability Score is a validated tool that predicts donor lung utilization and post-transplant recipient outcomes, but integration into practice remains limited. This study will evaluate determinants of LUNDON score adoption, its impact on clinical decision-making, and develop tools to support its implementation.

**Methods:**

This is a multi-aim mixed-methods pre-implementation study that will be conducted across U.S. Organ Procurement Organizations (OPOs) and lung transplant centers. Aim 1 will identify barriers and facilitators of LUNDON score adoption through surveys (n = 242 participants) and interviews (n = 60 participants) of OPO and transplant providers. Aims 2 and 3 will evaluate the impact of the LUNDON score on donor management and organ acceptance using simulation-based experiments with OPO coordinators, OPO clinical leadership, transplant pulmonologists, and transplant surgeons (n = 120 total). Aim 4 will synthesize findings to develop and pilot implementation toolkits across three OPOs and three transplant centers. Two implementation frameworks [the Capability, Opportunity, and Motivation-Behavior (COM-B) model and the Theoretical Domains Framework (TDF)] will guide data collection and analysis, integrating quantitative and qualitative findings.

**Expected impact:**

Findings from this collaborative, nationally supported study involving partners from the United Network for Organ Sharing (UNOS) and the national lung transplant community will inform the development of toolkits to support LUNDON score adoption in clinical practice, with the goal of reducing variation in donor management and increasing donor lung utilization. Future studies will explore the effect of LUNDON score adoption on donor lung utilization and recipient outcomes.

## Introduction

Despite significant advances in transplantation science [1–3], a scarcity of donor organs limits access to life-saving transplants across the United States [4,5] and globally [1,6]. This gap is especially pronounced in lung transplantation [7–9], where stringent donor selection criteria [10] result in underutilization of potentially viable donor organs [11]. Despite recent advances in donor allocation reform [12], the national lung utilization rate (defined as the proportion of deceased donors whose lungs are ultimately transplanted) in 2024 was only 37% [13], which is markedly lower than for other organs (~66% kidneys; ~ 64% livers) [13]. A persistent shortage of donor lungs results in significant mortality on the lung transplantation waitlist [7,14]. Effort-intensive and costly approaches such as ex-vivo lung perfusion have not yielded a substantial increase in available donor lungs. Moreover, over 40% of donor lungs currently declined may actually be suitable for transplantation [15], perhaps attributable to wide variability in donor lung procurement and acceptance practices [16,17] leading to inequities [18–23] and missed opportunities for transplantation [9,24,25].

### Lung transplantation process

Lung transplantation is a complex, highly coordinated, multistep process involving multiple key constituent groups. After a patient with advanced lung disease is listed for transplantation, donor identification and assessment begin. Donors may be declared after brain death (DBD) or after controlled circulatory death (DCD). Donor suitability is assessed using standard or extended criteria [11,25], and then donors are actively managed to optimize organ function [11]. During this period, donor lungs are vulnerable to injury from ventilator-associated lung injury [11,26,27], aspiration and pulmonary infection [11,26], chest trauma related to the cause of death and/or resuscitation maneuvers [11], and fluid overload [26,28]. These vulnerabilities, combined with conservative acceptance thresholds, contribute to the low national lung utilization rate [29]. Donor lung evaluation requires integration of clinical data while balancing recipient factors and logistical considerations.

Organ procurement organizations (OPOs) coordinate donor evaluation, management, and organ allocation, thereby serving as the critical link between donor hospitals and transplant centers. Variation in donor management practices (e.g., ventilation parameters, diuresis, bronchoscopy, and treatment of infection) can substantially influence whether lungs are ultimately transplanted [17,30–34]. Following donor management, donor lungs are offered to a potential recipient on the waitlist according to established allocation policies that integrate disease severity, comorbidities, and predicted benefit of transplantation [7,11,35]. The potential recipient’s transplant team then decides whether to accept the offer, influenced by center-specific thresholds, provider experiences, and recipient acuity. Once donor lungs are allocated, a specialized procurement team retrieves and transports them to the recipient hospital, where the transplantation surgery is performed, completing the process from listing to implantation.

Transplant providers make several critical decisions along this pathway that directly influence organ utilization. They are often made under time substantial time constraints related to donor stability, organ allocation logistics, and recipient readiness. Given this inherent complexity and variability, adoption of standardized, data-driven tools is essential to promote consistent, evidence-based decision-making in donor evaluation and organ acceptance.

### Lung donor (LUNDON) acceptability score

To address the need for more standardized, evidence-based tools for lung donor evaluation, our group previously developed and validated the Lung Donor (LUNDON) Acceptability Score [33], a predictive model that estimates the likelihood of donor lung utilization based on nine clinically relevant variables (**Table 1**) and correlates with post-transplant recipient outcomes [34]. Using a large national cohort from the Scientific Registry of Transplant Recipients (SRTR), we derived and validated the model in over 80,000 brain-dead donors. The LUNDON score demonstrates excellent discrimination and calibration across diverse donor populations [36,37]. The score is operationalized as a user-friendly, web-based tool (https://sites.wustl.edu/lundon/) that provides real-time case-specific probability estimates and supports more objective and reproducible decision-making in donor evaluation and management.

**Table 1:** LUNDON score component variables.

NON-MODIFIABLE VARIABLES	POTENTIALLY MODIFIABLE VARIABLES *(with possible donor management strategies for improvement)*
**• Donor age in years** **• Smoking history >20 pack-years (yes/no/unsure)** **• History of myocardial infarction (yes/no/unsure)** **• Mechanism of death by asphyxiation or drowning (yes/no)** • **Cardiac arrest after brain death (yes/no)**	**• PaO2/FiO2 ratio** *Lung protective ventilation*^15^ *Lung recruitment maneuvers*^15^ *Prone positioning*^37^ *Bronchoscopy*^15^ **• Abnormal chest x-ray appearance (yes/no)** *Frequent bronchoscopy* *Lung recruitment maneuvers* **• Maximum creatinine (mg/dL)** *Goal-directed fluid resuscitation*^38^ *Avoidance of nephrotoxic medications* *Renal replacement when indicated*^39^ *Hemodynamic optimization*^38^ **• Bloodstream infection (yes/no)** Prevention: *Isolation precautions, hand hygiene, sterile protocol for central line management* [40] Treatment: *Antibiotics, identification and drainage of infection source when indicated*

Four of the nine component variables in the LUNDON score are potentially modifiable (**Table 1**), including (1) donor PaO2/FiO2 [P/F] ratio, (2) chest x-ray appearance, (3) creatinine, and (4) bloodstream infection. Prior work by our group has shown that lung-protective ventilation strategies and goal-directed donor management can improve oxygenation, radiographic findings, and renal function, corresponding with higher organ utilization rates [17,38,39,41]. These findings indicate that targeted, evidence-based interventions can improve LUNDON scores and expand the donor lung pool, providing actionable targets for OPOs to enhance donor management practices.

### Implementation gap

Despite strong validation across multiple studies, the LUNDON score remains underutilized in routine donor management and transplant decision-making. This reflects a broader and well-recognized phenomenon in medicine, where the translation of evidence-based innovations into routine practice is slow, inconsistent, and often incomplete [16,42,43]. Predictive tools such as the LUNDON score demonstrate excellent discrimination for donor lung utilization and post-transplant recipient outcomes, yet their integration into the time-sensitive, high-stakes clinical environment of transplantation remains limited.

Barriers to implementation of evidence-based practices in lung transplantation, such as the LUNDON score, may occur at multiple levels [44,45]. At the individual-level donor management decisions are frequently shaped by subjective judgment [46–49], institutional norms [46,50–52], and provider experience [46,50] rather than standardized data-driven approaches [11]. At the organizational level, variability in OPO workflows, limited interoperability of data systems, and the absence of embedded decision-support tools constrain adoption [8,53–55]. At the system level, the absence of structured implementation frameworks and performance feedback mechanisms perpetuates variation in donor evaluation and acceptance practices across centers [56,57].

Recent studies reinforce the need for deliberate implementation efforts. Our group and others have demonstrated that while the LUNDON score reliably predicts donor lung recovery and post-transplant recipient outcomes, its effectiveness depends on local calibration, consistent donor management strategies, and organizational readiness for change [34,36,37]. Collectively, these findings underscore that predictive validity alone is insufficient to drive behavioral change in the lung transplantation workflow.

### Implementation science

Implementation science is a field dedicated to promoting the adoption and sustained use of evidence-based practices [58] across diverse healthcare contexts through structured, multi-level frameworks and methods [59]. These frameworks help identify barriers and facilitators, develop targeted strategies, and evaluate mechanisms that drive behavioral and organizational change. Applying implementation science principles in the context of lung donor management [16,60] offers an opportunity to accelerate the integration of predictive models such as the LUNDON score into routine clinical practice.

### Expected impact

Grounded in implementation science methods, the proposed study aims to identify determinants (barriers and facilitators) of LUNDON score adoption in OPOs and lung transplant centers.

The integration of the LUNDON score into routine clinical practice has the potential to standardize donor evaluation, reduce unwarranted practice variation, and expand the pool of transplantable lungs. Ultimately, findings from this study will inform a future hybrid effectiveness–implementation trial designed to assess the real-world impact of LUNDON-guided donor management on organ utilization and recipient outcomes.

## Methods/Design

*Aims:*

- **Aim 1**: Identify determinants (barriers and facilitators) of LUNDON score adoption.
- **Aim 2**: Evaluate the LUNDON score’s impact on donor management at OPOs.
- **Aim 3**: Evaluate the LUNDON score’s impact on donor lung acceptance decisions at lung transplant centers.
- **Aim 4**: Develop and pilot implementation toolkits to support national dissemination and uptake of the LUNDON score.

**Fig 1:**
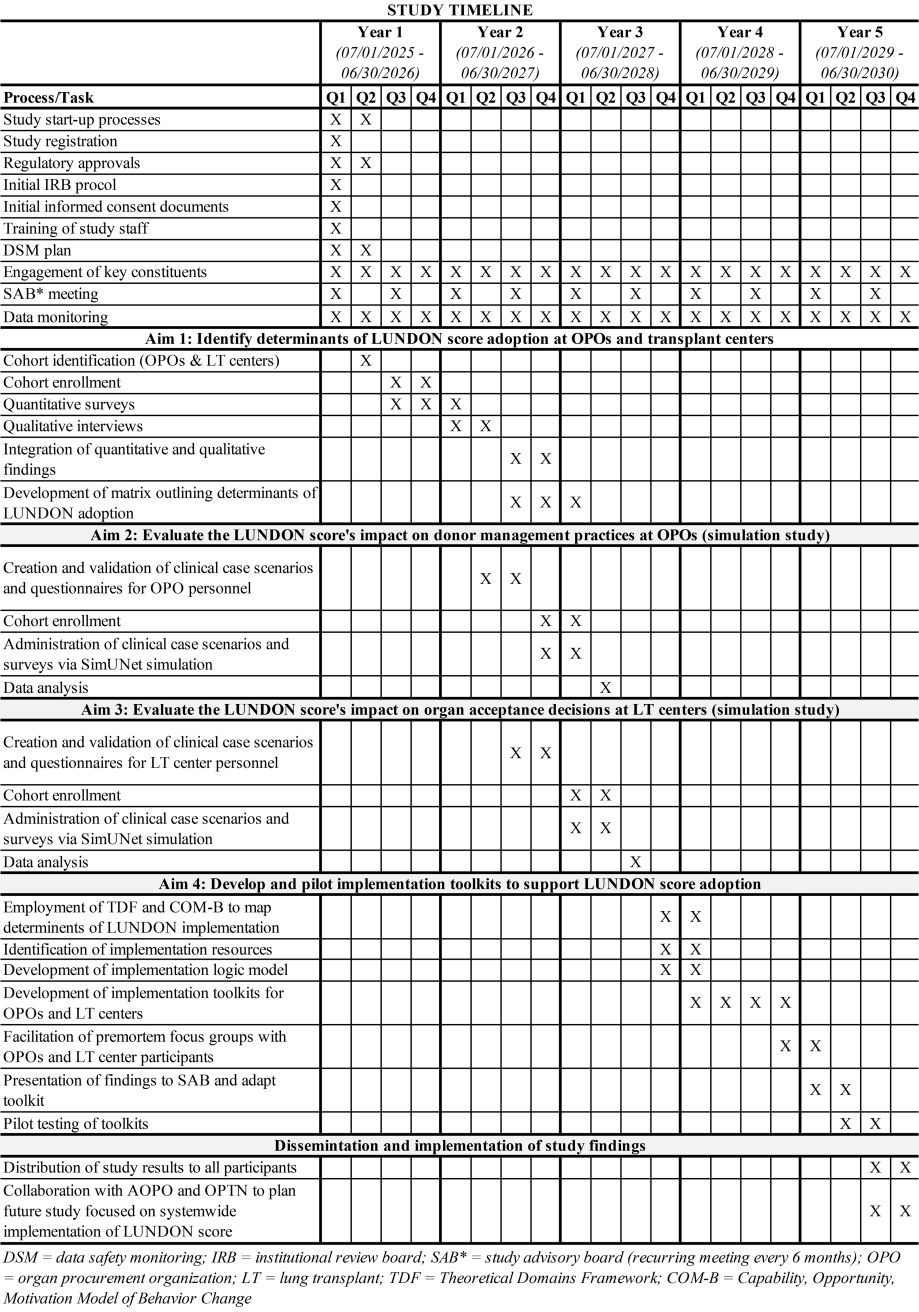
Study timeline and key activities.

*Timeline:* The complete study timeline is provided in Fig 1. At the time of manuscript submission, participant recruitment has not yet been completed for any study aim, and no study results have been generated. Recruitment and data collection will proceed in staged phases as outlined in the study timeline (Fig 1). At the time of protocol submission, no study results were available.

Participant recruitment for Aim 1 is expected to begin on 01/02/2026 and continue through 30/06/2027. Recruitment for Aims 2 and 3 is expected to occur between 01/03/2027 and 31/12/2027. Recruitment for Aim 4 pilot testing is expected to occur between 01/09/2029 and 30/06/2030.

Based on the current study timeline, we estimate that participant recruitment will be completed by 30/06/2030 and data collection will be completed by 31/08/2030. As this is a multiple aim study with sequential phases, analyses and data dissemination will occur after completion of recruitment and data collection for each aim.We expect primary results beginning in 2027 and continuing through the end of the study period in 2030.

### Study design and setting

This is a pre-implementation study that will focus on U.S. OPOs and lung transplant centers, the key environments where donor evaluation and organ acceptance decisions occur.

### Study participants

Participants (**Table 2**) will include the following key constituent groups that are directly involved in donor evaluation and acceptance decisions: (1) OPO clinical leadership, (2) OPO donor coordinators, (3) transplant pulmonologists, and (4) transplant surgeons.

**Table 2:** Overview of target sample populations.

	AIM 1		AIM 2	AIM 3	AIM 4	
	Quantitative Phase	Qualitative Phase			Refinement Phase	Evaluation Phase
**Sample Population**	National sample from OPOs and LT centers	Purposive sample* of survey respondents	Purposive sample* of OPO personnel	Purposive sample* of LT providers	Key constituents from partner sites (3 OPOs, 3 lung transplant centers)	
**Sample Size**	N = 242	N = 60	N = 60	N = 60	N = 48	N = 12
**Participants**	OPO personnel (n = 112), LT providers (n = 130)	OPO personnel (n = 30), LT providers (n = 30)	CMOs (n = 30), Coordinators (n = 30)	Surgeons (n = 30), Pulmonologists (n = 30)	CMOs (n = 12), Coordinators (n = 12), Surgeons (n = 12), Pulmonologists (N = 12)	CMOs (n = 3), Coordinators (n = 3), Surgeons (n = 3), Pulmonologists (n = 3)
**Consent Process**	Implied by survey completion	Formal written	Implied by survey completion	Implied by survey completion	Formal written	Formal written

*Purposive sampling will ensure representation from low-, intermediate-, and high-performing organ procurement organizations (OPOs) and lung transplant (LT) centers (as defined by publicly reported lung utilization rates and organ acceptance rates, respectively).

These multidisciplinary groups represent the primary potential end-users of the LUNDON score and are integral to understanding contextual barriers and facilitators to its adoption. Participants will be identified through the professional networks of the study team and invited with support from the study’s advisory board to ensure broad representation across institutions and geographic regions.

### Sample size determination and sampling strategy

Sample sizes for each aim are detailed below. Sampling strategies were informed by our team’s prior unpublished work within the relatively small, close-knit community of OPOs and lung transplant clinicians. Based these experiences, we anticipate response rates exceeding 70%. Sample size estimates account for this expected level of participation.

To evaluate and minimize potential non-response bias, an interim analysis will be conducted after 40% of the survey samples have been enrolled for Aims 1–3. Key demographic and professional characteristics of responders and non-responders will be compared. If systematic differences are observed, targeted oversampling will be employed to ensure representation of participants with characteristics similar to non-responders for the remainder of enrollment.

### Implementation frameworks guiding the study

This study applies two complementary implementation science frameworks to guide the identification of determinants of LUNDON score usage and the development of implementation toolkits to promote adoption:

1) The Capability, Opportunity, Motivation-Behavior (COM-B) model [16,61] posits that behavior is driven by three essential components: capability (knowledge and skills), opportunity (environmental and social factors), and motivation (automatic and reflective processes). This model provides a structured lens for conceptualizing why specific behaviors related to donor management and organ acceptance occur (or fail to occur) in real-world practice.
2) The Theoretical Domains Framework (TDF) [62] consolidates multiple behavior change theories into 14 domains to help systematically identify barriers (factors that impede implementation) and facilitators (factors that promote implementation). The framework supports the selection of tailored strategies to address the identified determinants of behavior.

Together, these frameworks will be used to: (a) identify barriers and facilitators of LUNDON score adoption in clinical practice and map the corresponding behavioral domains [Aim 1]; (b) characterize the behavioral and contextual factors influencing OPO-level donor management decisions related to modifiable LUNDON score variables [Aim 2]; and (c) assess how knowledge of a donor’s LUNDON score and the corresponding estimate of recipient survival influences organ acceptance decisions at the lung transplant center level [Aim 3].

### Aim-Specific Protocols and Data Collection

#### Aim 1: Identify determinants (barriers and facilitators) of LUNDON score adoption.

We will conduct a sequential explanatory mixed-methods study consisting of a national survey (quantitative phase, n = 242) followed by semi-structured interviews (qualitative phase, n = 60). Sample size estimates are guided by the concept of “information power”, which indicates that more specialized participant insight allows for smaller sample sizes. Information power is affected by the study aim, the specificity of participant experiences, the guiding theoretical frameworks (COM-B [16, 61] and TDF [62]), anticipated data quality, and analytic strategy.

In the quantitative phase, we will administer a survey designed to understand providers’ capability, motivation and opportunities around lung donation and acceptance processes. Participants will occur via email invitation, with up to three reminders. Informed consent will be implied by survey completion after review of the study information sheet. After answering demographic questions and some broader questions about their donor management practices, participants will view a brief educational video introducing the LUNDON score. Participants will then answer questions about their interest in adopting the LUNDON score in their clinical practice. Survey instruments will be tailored for OPO representatives and lung transplant providers, respectively.

In the qualitative phase, we will conduct 45-minute semi-structured interviews with a purposive sample of survey respondents across high-, middle-, and low-performing OPOs and lung transplant centers based on publicly reported lung utilization and offer acceptance rates. Participants will provide written electronic consent before their interview. Interview guides, informed by the COM-B and the TDF frameworks, will be tailored to OPO and lung transplant provider roles and focus on behavioral drivers, contextual barriers and facilitators, and the perceived feasibility of LUNDON score adoption. Interviews will be conducted virtually via Zoom™, audio-recorded, transcribed verbatim, and thematically analyzed using NVivo™. Findings will be triangulated with survey results through a mixed-methods matrix to identify high-priority, modifiable implementation strategies aiming to support the development of resources in Aim 4.

### Aims 2 and 3: Assess the impact of the LUNDON score availability on clinical decision making related to donor management [among OPO representatives] and organ offer acceptance [among lung transplant providers]

We will recruit OPO personnel (Aim 2, N = 60) and lung transplant providers (Aim 3, N = 60) to participate in a simulation-based experiment designed to evaluate how access to the LUNDON score influences clinical decision-making. Purposive sampling will ensure representation from low-, medium-, and high-performing OPOs and lung transplant centers. Participants will be recruited via email invitation, with up to three reminders. Informed consent will be implied by initiation of the simulation activity and subsequent survey completion following review of the study information sheet.

Simulation activities will be conducted in SimUNet, a web-based platform developed by the United Network for Organ Sharing (UNOS) [63] that mimics real-time clinical workflows in DonorNet, the official system used by the Organ Procurement and Transplantation Network (OPTN) for management of donor information and organ offers. UNOS developed and maintains DonorNet as the OPTN contractor and created SimUNet for research and experimental purposes. SimUNet has been validated in national studies assessing kidney offer decisions, demonstrating both feasibility and realism for simulating transplant workflows [64,65].

After orientation to SimUNet, participants will view a brief educational video introducing the LUNDON score and its potential applications for lung donation and then complete 16 simulated case scenarios representing a range of donor risk profiles and clinical complexity. The order of scenario presentation and the visibility of the LUNDON score will be randomized to reduce order effects and individual bias. For each case, OPO personnel will provide donor management recommendations, while lung transplant providers will accept or decline organ offers; all participants will rate their decisional confidence and provide open-text justifications.

Quantitative data will be analyzed using generalized estimating equations to model within-subject differences in decision-making between scenarios with and without the LUNDON score. Prespecified subgroup analyses will assess heterogeneity by transplant center volume and by Lung Composite Allocation Score (CAS), a metric used by OPTN to assess transplant center performance based on waitlist, transplant, and post-transplant outcomes [12].

#### Aim 4: Develop and pilot-test implementation toolkits for LUNDON score adoption.

**Fig 2:**
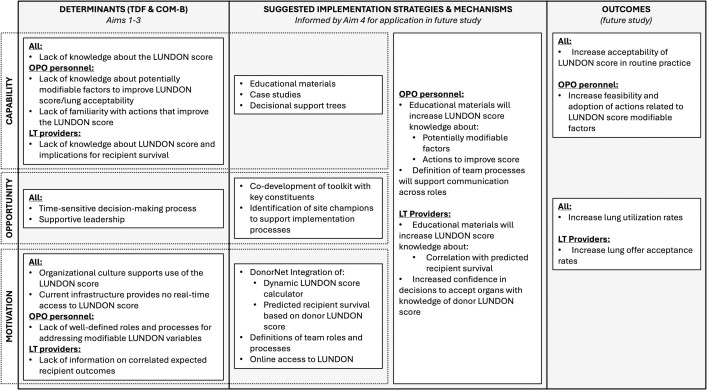
Proposed implementation research logic model that will be adapted to incorporate findings from Aims 1-3.

Based on the findings from Aims 1–3, we will follow a structured process [66] to develop a set of participant-informed implementation toolkits to support real-world use of the LUNDON score. This will proceed in three phases:

• Phase 1: Develop an Implementation Research Logic Model (IRLM), to graphically depict relationships among study elements, aid future study replicability, and evaluate the implementation process [67]. A proposed IRLM for this study is provided in **Fig 2**. In Aim 4, the IRLM will be revised to incorporate findings from Aims 1–3, using the COM-B model and the TDF to systematically map identified behavioral determinants of LUNDON score adoption. The research team will then link these determinants to corresponding implementation strategies and potential toolkit resources.

- Phase 2: Develop tailored toolkits for OPOs and lung transplant centers, comprised of resources to address the site-specific contextual needs for LUNDON score implementation. Guided by COM-B and TDF, toolkit development will draw on findings from Aims 1–3, clinical experience, and the input of multiple key constituent groups. Toolkit components may include decision-support aids, brief user training modules, electronic health record integration templates, and/or communication scripts tailored to clinical workflows.
- Phase 3: Refine toolkits through a brainwriting [68] pre-mortem exercise [69], which is a proactive planning method that uses prospective hindsight to anticipate potential reasons for implementation failure and generate solutions before the intervention is deployed. Focus groups will engage key constituents (n = 48) to assess content, format, acceptability, and usability, and will proactively identify weaknesses, barriers to adoption, and potential points of failure in the toolkit design. Participants will be offered a $125 Visa gift card as compensation.
- Phase 4: Pilot test the toolkits at three OPOs and three lung transplant centers. Pilot testing will include brief user testing followed by structured interviews with decision-making personnel at each site (n = 12) to assess acceptability, feasibility, and suggestions for improvement. Qualitative data will inform iterative refinement of materials. Interview participants will be offered a $125 Visa gift card as compensation.

### Safety considerations

This is a minimal-risk study. The primary potential risks is a breach of confidentiality, which will be mitigated through use of secure data platforms and IRB-approved data protection procedures. No biomedical interventions or physical risks are associated with participation.

### Data management

All study data will be managed using secure, access-controlled platforms in compliance with institutional policies and relevant federal regulations. Each participant will be assigned a unique study ID, and all personally identifiable information will be stored separately from analytic datasets. Data access will be restricted to approved study team members.

Survey data (Aims 1–3) will be collected and stored in REDCap. Interview recordings will be transcribed verbatim by a secure transcription service and uploaded to NVivo for qualitative coding and thematic analysis.

Routine data quality checks will be conducted weekly to identify missing or inconsistent entries. Periodic data backups will be stored on encrypted institutional servers. Final analytic datasets will be de-identified, with documentation maintained for transparency and reproducibility. Following study completion, data will be made available to qualified researchers upon request and IRB approval.

### Ethics approval and consent to participate

This study protocol has been reviewed and approved by the Institutional Review Boards (IRBs) of Washington University [IRB # 202504071] and Duke University [IRB # Pro00118530]. All participants will provide informed consent prior to participation.

### Dissemination plan

Study findings will be disseminated through peer-review publications, including this protocol and subsequent results manuscripts. Results will also be presented at national conferences focused on transplantation and implementation science. Upon study completion, finalized implementation toolkits will be made freely available through an open-access study website and shared with the broader transplant community via UNOS and professional society networks to support real-world application.

## Discussion

Despite substantial progress in donor management and organ allocation, lung utilization in the United States remains low, with wide variation across centers and persistent gaps between evidence and practice. The LUNDON score offers a validated, data-driven approach to predict donor lung utilization and post-transplant outcomes; however, its integration into routine donor management and clinical decision-making has been limited. This study addresses a critical gap in the literature by applying implementation science methods to identify behavioral, organizational, and contextual determinants of LUNDON score adoption and by developing actionable, theory-informed strategies to promote its routine use.

This work represents a collaborative, nationally supported effort involving partners from the United Network for Organ Sharing (UNOS), the surgical and medical lung transplant community, and OPOs. The study rationale, design, and proposed interventions have been shaped by direct input from these constituent groups, ensuring alignment with clinical practice realities and system-level priorities. By systematically identifying barriers and facilitators and translating these findings into practical implementation tools, this study aims to bridge the evidence-to-practice gap and improve donor lung utilization nationwide.
