# Correction: Implementing the Lung Donor (LUNDON) acceptability score in U.S. donor management and transplant decision-making: A multi-aim, mixed-methods protocol

**DOI:** 10.1371/journal.pone.0353279

**Published:** 2026-07-06

**Authors:** Nikki E. Rossetti, Samantha Morrison, Su-Hsin Chang, Yan Yan, Graham Jaensch-Frie, Charles R. Liu, Nahom Y. Seyoum, Meghna Katta, Arvind Kumar, Zhizhou Yang, Christian Oncken, Christy Hamilton, Nicholas Schroy, Kyle Stumbaugh, Gary Marklin, Brendan Heiden, Brendon Cummiskey, Sarah Peskoe, Matthew Hartwig, Varun Puri, Ana A. Baumann

There are errors in the Author Contributions. The correct contributions are:

**Conceptualization:** Nikki E. Rossetti, Samantha Morrison, Su-Hsin Chang, Yan Yan, Gary Marklin, Brendan Heiden, Brendon Cummiskey, Sarah Peskoe, Varun Puri, Matthew Hartwig, Ana A. Baumann.

**Data curation:** Nikki E. Rossetti, Christian Oncken.

**Funding acquisition:** Matthew Hartwig, Varun Puri, Ana A. Baumann.

**Investigation:** Brendon Cummiskey, Sarah Peskoe, Matthew Hartwig, Ana A. Baumann, Varun Puri

**Methodology:** Nikki E. Rossetti, Samantha Morrison, Su-Hsin Chang, Yan Yan, Charles R. Liu, Nahom Y. Seyoum, Meghna Katta, Arvind Kumar, Gary Marklin, Brendon Cummiskey, Sarah Peskoe, Matthew Hartwig, Varun Puri, Ana A. Baumann.

**Project administration:** Nikki E. Rossetti, Graham Jaensch-Frie, Christy Hamilton, Kyle Stumbaugh

**Software:** Nicholas Schroy, Brendon Cummiskey.

**Supervision:** Brendon Cummiskey, Sarah Peskoe, Matthew Hartwig, Varun Puri, Ana A. Baumann

**Validation:** Zhizhou Yang, Brendan Heiden.

**Visualization:** Nikki E. Rossetti.

**Writing – original draft:** Nikki E. Rossetti.

**Writing – review & editing:** Nikki E. Rossetti, Samantha Morrison, Su-Hsin Chang, Yan Yan, Charles R. Liu, Nahom Y. Seyoum, Meghna Katta, Arvind Kumar, Zhizhou Yang, Gary Marklin, Brendan Heiden, Brendon Cummiskey, Sarah Peskoe, Matthew Hartwig, Varun Puri, Ana A. Baumann.
